# Marked Third-Trimester Placental Thickening Is Associated with Maternal Infectious, Hormonal, and Metabolic Burden: A Matched Case-Control Study

**DOI:** 10.3390/jcm15155990

**Published:** 2026-08-01

**Authors:** Julia Murlewska, Maria Respondek-Liberska, Iwona Strzelecka

**Affiliations:** 1 Department of Prenatal Cardiology, Polish Mother’s Memorial Hospital Research Institute, 93-338 Lodz, Poland; 2 Department for Fetal Malformations Diagnoses & Prevention, Faculty of Public Health, Medical University of Lodz, 90-419 Lodz, Poland

**Keywords:** placental thickness, thick placenta, case–control study, maternal infection, hypothyroidism, gestational diabetes mellitus, maternal obesity, fetal dysfunction, prenatal ultrasound

## Abstract

**Background:** Increased placental thickness has been associated with adverse perinatal outcomes and fetal functional and structural abnormalities. However, whether marked third-trimester placental thickening is associated with a distinct maternal clinical profile compared with pregnancies with normal placental thickness remains insufficiently characterized. This study aimed to compare maternal characteristics, comorbidities, medication exposure, infection history, and fetal findings between pregnancies with marked placental thickening, defined as placental thickness ≥70 mm, and gestational-age-matched control pregnancies with a placental thickness <70 mm and no documented maternal or fetal abnormalities. **Methods:** This retrospective matched case–control study included singleton pregnancies referred for fetal echocardiography to a tertiary referral center in Łódź, Poland, between 1 January 2022 and 14 March 2025. Placental thickness was measured sonographically in a perpendicular plane from the chorionic plate to the basal plate, excluding the umbilical cord insertion site. Only anterior and/or fundal placentas assessed at ≥28 weeks of gestation were included. Among pregnancies with recorded third-trimester placental thickness measurements, 99 cases with placental thickness ≥70 mm were identified as the thick-placenta group. A control group of 99 pregnancies with placental thickness <70 mm was selected and matched for gestational age. Control pregnancies had no documented maternal disease, no fetal structural or functional abnormalities, and no exposure to the medications analyzed in this study. Maternal demographic characteristics, body mass index, comorbidities, infection history, obstetric history, and medication use were compared between groups. Continuous variables were compared using Welch’s t-test and the Mann–Whitney U test, and categorical variables were compared using Fisher’s exact test. **Results:** The study included 99 pregnancies with marked placental thickening and 99 control pregnancies with normal placental thickness. Gestational age at examination was comparable between groups, with a mean of 35.5 weeks in controls and 35.0 weeks in the thick-placenta group (*p* = 0.662, Welch’s *t*-test). Median gestational age was also not significantly different between groups (35.4 vs. 36.43 weeks; *p* = 0.340, Mann–Whitney U test). Mean placental thickness was significantly greater in the thick-placenta group than in controls (81.4 mm vs. 46.9 mm; *p* < 0.0001). Maternal age and anthropometric characteristics were comparable between groups, whereas BMI > 25 kg/m^2^ was more common in the thick-placenta group. In contrast to the clinically healthy control group, maternal infection was documented in 100.0% of thick-placenta cases, hormonal treatment in 97.0%, history of COVID-19 in 52.5%, hypothyroidism in 44.4%, prior miscarriage in 37.4%, aspirin or anticoagulant use in 32.3%, gestational diabetes mellitus in 25.3%, and pregnancy-induced hypertension in 7.1%. All evaluated maternal clinical factors were significantly more common in the thick-placenta group than in controls. Fetal cardiac or extracardiac dysfunction was present in 68.7% of thick-placenta pregnancies. **Conclusions:** In this gestational-age-matched case–control study, pregnancies with marked third-trimester placental thickening showed a distinct maternal profile compared with healthy controls with normal placental thickness. Despite comparable gestational age, maternal age, and maternal anthropometric characteristics, the thick-placenta group demonstrated a significantly higher infectious, hormonal, metabolic, and endocrine burden. These findings indicate that, in this selected tertiary referral cohort, placental thickness ≥70 mm was associated with a higher burden of maternal clinical abnormalities and fetal functional findings. Rather than representing an independent marker of placental maladaptation or maternal-fetal risk, marked placental thickening should be interpreted as a clinically relevant ultrasound finding that may prompt careful review of maternal history and targeted fetal assessment. Prospective studies are needed to determine which maternal factors are independently associated with placental thickening and to clarify their relationship with fetal function and perinatal outcomes.

## 1. Introduction

Our previous study [1] demonstrated a significant association between increased placental thickness and fetal functional abnormalities, particularly cardiac and extracardiac dysfunctions, even in the absence of overt structural malformations. However, that analysis focused primarily on fetal findings and did not fully address the maternal clinical background associated with abnormal placental thickening.

In the present follow-up investigation, we evaluated whether marked third-trimester placental thickening is associated with a distinct maternal profile when compared with pregnancies with normal placental thickness. We therefore compared 99 singleton pregnancies with a placental thickness ≥70 mm with 99 gestational-age-matched control pregnancies with a placental thickness <70 mm. The control group included clinically healthy pregnant patients without documented maternal disease, without fetal structural or functional abnormalities, and without exposure to the medications evaluated in this study.

By comparing pregnancies with marked placental thickening to a matched healthy control group, we aimed to clarify whether excessive placental thickness is associated with maternal infectious, hormonal, metabolic, and endocrine burden independently of gestational age at examination.

## 2. Materials and Methods

### 2.1. Study Population and Methodology

This was a retrospective, gestational-age-matched case–control study conducted to evaluate maternal factors associated with marked placental thickening in the third trimester of pregnancy. The study was based on fetal echocardiographic and obstetric ultrasound data collected at the Department of Prenatal Cardiology, Polish Mother’s Memorial Hospital Research Institute, a tertiary referral center in Łódź, Poland, between 1 January 2022 and 14 March 2025.

During the study period, 2082 fetal echocardiograms were performed. Referrals were primarily related to abnormal screening ultrasound findings, maternal disease or comorbidity, and family history of congenital heart disease. Placental thickening itself was not an isolated indication for referral (Table 1); placental thickness was assessed during the targeted fetal and echocardiographic examination performed at the tertiary referral center.

Placental thickness was assessed during the same targeted fetal ultrasound and echocardiographic examination. Measurements were obtained in a perpendicular plane from the chorionic plate to the basal plate, avoiding the umbilical cord insertion site, placental edges, focal placental lesions, and tangential sections. The myometrium and retroplacental space were not included in the measurement.

For each case, several measurements were obtained in the visually thickest representative part of the placenta, and the final placental thickness value used for analysis was the mean of these repeated measurements. If more than one placental region was assessed, the highest representative mean value was recorded as the final placental thickness.

Measurements were performed by one of two experienced operators during routine clinical assessment. Because of the retrospective nature of the study, not all cases were independently measured by both operators. Operators were not formally blinded to the clinical referral indication or available maternal history at the time of examination; however, final study-group allocation was performed retrospectively based on the recorded placental thickness threshold.

Formal intraobserver and interobserver variability analyses were not performed and are acknowledged as a methodological limitation.

Among 1452 pregnancies with recorded third-trimester placental thickness measurements, 99 cases with a placental thickness ≥70 mm were identified and included in the thick-placenta group. A control group of 99 singleton pregnancies with a placental thickness <70 mm was selected from the available database and matched to the thick-placenta group for gestational age at examination. Control pregnancies were required to have no documented maternal disease, no fetal cardiac or extracardiac structural or functional abnormality, and no exposure to the medications analyzed in this study, including hormonal treatment, aspirin, or anticoagulants. All control placentas were located on the anterior uterine wall and/or uterine fundus.

**Figure 1:**
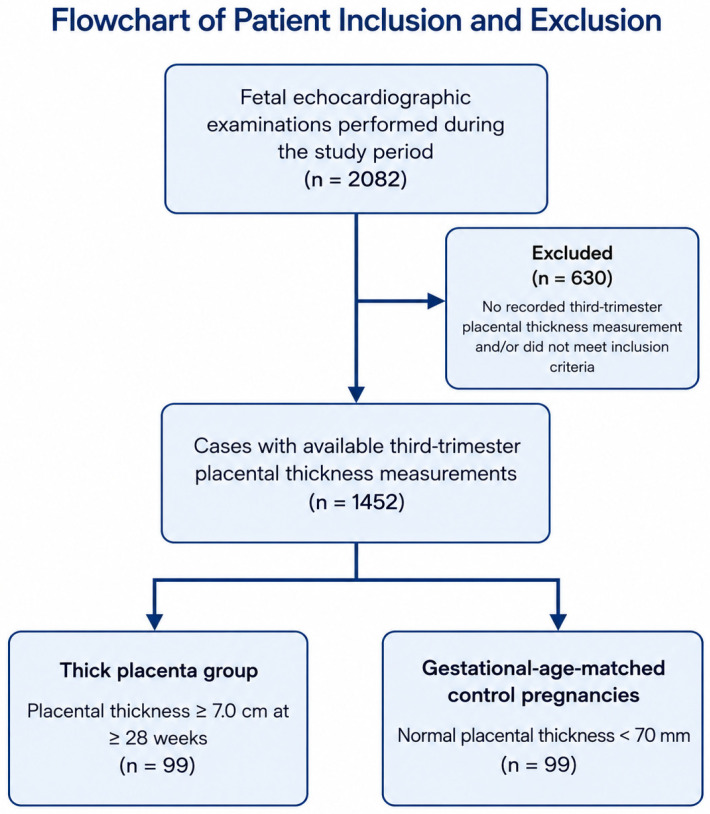
Patient inclusion and exclusion flowchart. Of 2082 fetal echocardiographic examinations performed during the study period, 1452 cases had third-trimester placental thickness measurements and were included in the analysis. The thick-placenta group included 99 pregnancies, and 99 gestational-age-matched pregnancies with normal placental thickness served as controls. Neonatal outcome data were available for 27 cases from the thick-placenta group.

The final study population therefore consisted of 198 singleton pregnancies: 99 pregnancies with marked placental thickening and 99 gestational-age-matched controls with normal placental thickness. Neonatal outcome data were available for a subset of 27 cases from the thick-placenta group and included gestational age at delivery, birth weight, Apgar scores, mode of delivery, preterm birth, respiratory failure requiring NICU care, neonatal jaundice, transient pulmonary hypertension, and neonatal death. Placental weight was not available (Figure 1). Owing to the retrospective design and anonymized data analysis, the requirement for approval by the institutional bioethics committee of the Polish Mother’s Memorial Hospital Research Institute was waived. The manuscript was prepared in accordance with the STROBE reporting recommendations for observational studies.

### 2.2. Maternal Data Collection

For each patient, data were collected from a standardized clinical intake questionnaire (Table 2) and available medical documentation. The questionnaire included maternal age, pre-pregnancy weight and height, weight at the time of examination, current and past medical history, obstetric history, infection history, and medication exposure during pregnancy.

Maternal comorbidities evaluated in the study included gestational diabetes mellitus, pregnancy-induced hypertension, hypothyroidism, prior miscarriage, and prior or active infections. Medication exposure included aspirin, anticoagulants such as Clexane, and hormonal progesterone support, including Duphaston, Utrogestan, Luteina, and progesterone preparations. Nutritional supplementation was recorded but was not analyzed as a disease-related medication exposure.

Infection-related findings during the index pregnancy were recorded using a structured approach rather than as a single homogeneous diagnosis. These findings included documented symptomatic infection, asymptomatic colonization or positive microbiological screening, serological evidence of infectious exposure, PCR-confirmed infection, and questionnaire-reported infectious history.

For each infection-related finding, the following variables were recorded whenever available: pathogen type, pathogen group, anatomical site, trimester or date of diagnosis, presence or absence of symptoms, clinical symptoms, diagnostic method, and treatment. Pathogen groups were categorized as bacterial, fungal, viral, or parasitic. Anatomical sites were categorized as genitourinary, respiratory, gastrointestinal, systemic, or other.

The diagnostic basis was classified as urine culture, vaginal or cervical swab, other microbiological culture, PCR testing, serology, medical-record-based clinical diagnosis, or standardized questionnaire report. Questionnaire-reported infectious history was recorded as a separate data source and was not considered equivalent to microbiologically confirmed active infection unless supported by medical documentation or laboratory testing.

Therefore, the variable referred to as infection during pregnancy should be interpreted as a broad composite variable describing infection-related maternal findings during the index pregnancy. It includes both active infections and colonization/positive infectious screening results and was analyzed descriptively.

The control group was intentionally selected as a clinically healthy comparison group with normal placental thickness, no documented maternal disease, no fetal structural or functional abnormalities, and no exposure to the medications analyzed in this study. Therefore, the control group should not be interpreted as an unselected obstetric population. Rather, it served as a clearly defined healthy reference group to describe the maternal and fetal clinical profile associated with marked placental thickening under controlled retrospective conditions.

Because the absence of maternal disease, fetal abnormalities, and analyzed medication exposure was part of the control-group definition, between-group comparisons involving these variables were expected to show large differences by design. These comparisons were therefore interpreted descriptively and should not be regarded as unbiased estimates of population-level risk.

Controls were selected from the available database of pregnancies with normal placental thickness and were matched to the thick-placenta group according to gestational age at examination. The purpose of matching was to reduce the influence of gestational age on placental thickness because placental dimensions may vary across pregnancy. Control selection was performed retrospectively from eligible examinations with a comparable gestational age and anterior and/or fundal placental location.

### 2.3. Statistical Processing

The dataset was numerically coded and analyzed using descriptive and comparative statistics. Continuous variables were summarized as means, standard deviations, medians, and ranges, as appropriate. Categorical variables were summarized as counts and percentages.

Between-group comparisons were performed between the thick-placenta group and the gestational-age-matched control group. Welch’s *t*-test was used for comparison of continuous variables when appropriate, particularly when variances were unequal. The Mann–Whitney U test was used for non-parametric comparison of continuous variables. Fisher’s exact test was used for categorical variables, including maternal comorbidities, infection history, medication exposure, and pregnancy-induced hypertension. A two-sided *p*-value < 0.05 was considered statistically significant.

All between-group comparisons were performed as univariable analyses. Multivariable regression modeling was not performed because the study design used a highly selected healthy control group in which maternal disease, fetal abnormalities, and analyzed medication exposure were absent by definition. This resulted in complete or near-complete separation for several variables, including infection-related findings, hormonal treatment, hypothyroidism, gestational diabetes mellitus, aspirin/anticoagulant use, and pregnancy-induced hypertension. Under these conditions, multivariable logistic regression would be expected to produce unstable or non-estimable effect estimates. Therefore, the reported *p*-values should be interpreted as descriptive unadjusted comparisons between the thick-placenta group and the selected healthy control group, rather than as adjusted estimates of independent risk.

Gestational age matching was performed during control selection by choosing pregnancies with normal placental thickness and comparable gestational age at examination. The balance in gestational age between groups was summarized using descriptive statistics and compared using Welch’s *t*-test and the Mann–Whitney U test. The absence of a statistically significant between-group difference was not interpreted as the sole criterion for matching adequacy.

Exclusion criteria included multifetal pregnancy, chromosomal syndrome, known placenta previa or low-lying placenta, posterior placental location, abrupted placental location, missing placental thickness measurement, and missing or incomplete questionnaire data.

## 3. Results

The final study population included 198 singleton pregnancies: 99 pregnancies with marked third-trimester placental thickening, defined as placental thickness ≥70 mm, and 99 gestational-age-matched control pregnancies with a placental thickness <70 mm (Table 3). All placentas were located on the anterior uterine wall and/or uterine fundus. All *p*-values presented in Table 3 represent univariable, unadjusted comparisons and should be interpreted descriptively in the context of the selected control-group design.

Gestational age at examination was comparable between groups. The mean gestational age was 35.5 weeks in the control group and 35.0 weeks in the thick-placenta group, with no statistically significant difference between groups (*p* = 0.662, Welch’s *t*-test). Median gestational age was also similar between groups: 35.4 weeks in controls and 36.43 weeks in the thick-placenta group (*p* = 0.340, Mann–Whitney U test). The gestational age range was 33.4–38.4 weeks in the control group and 28–40 weeks in the thick-placenta group.

As expected by study design, placental thickness differed significantly between groups. Mean placental thickness was 46.9 mm in controls and 81.4 mm in the thick-placenta group (*p* < 0.0001, Welch’s *t*-test). Median placental thickness was 45.7 mm in controls and 80.0 mm in the thick-placenta group. The placental thickness range was 27.3–68.6 mm in controls and 70.0–123.4 mm in the thick-placenta group.

Maternal age and anthropometric characteristics were comparable between groups. Mean maternal age was 31 years in controls and 31.2 years in the thick-placenta group. Mean maternal height was 162 cm in controls and 165 cm in the thick-placenta group, while mean maternal weight was 73 kg and 77.3 kg, respectively. Mean maternal BMI was 27.6 kg/m^2^ in controls and 28.4 kg/m^2^ in the thick-placenta group. BMI > 25 kg/m^2^ was present in 60% of controls and 75.0% of patients in the thick-placenta group.

**Figure 2:**
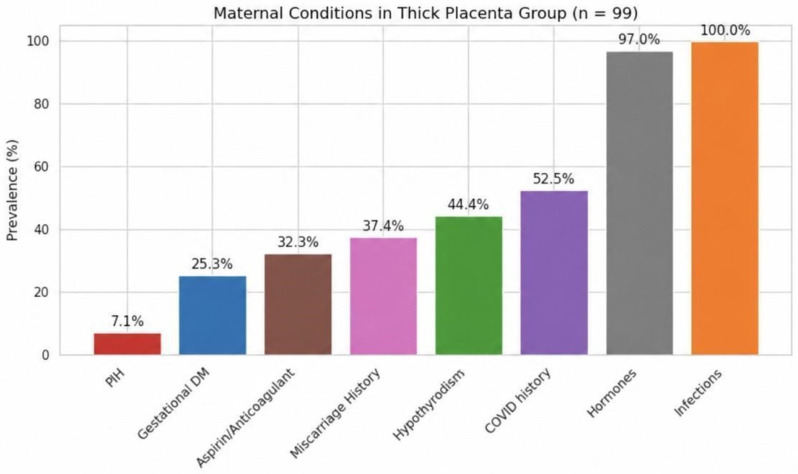
Bar chart showing maternal conditions in the thick-placenta group (n = 99), sorted from the least to most prevalent: PIH: 7.1%, Gestational DM: 25.3%, Aspirin/Anticoagulant intake: 32.3%, Miscarriage History: 37.4%, Hypothyroidism: 44.4%, COVID history: 52.5%, Hormonal treatment: 97.0%, Infections during the pregnancy: 100.0%.

The control group consisted of clinically healthy pregnant patients without documented maternal disease, infection, or exposure to the medications evaluated in this study. In contrast, pregnancies in the thick-placenta group showed a significantly higher burden of maternal clinical abnormalities. Infection during pregnancy was documented in 99/99 cases (100.0%) in the thick-placenta group and in 0/99 controls (*p* < 0.0001, Fisher’s exact test). Hormonal treatment during pregnancy was reported in 96/99 cases (97.0%) in the thick-placenta group and in 0/99 controls (*p* < 0.0001) (Figure 2).

Other maternal findings were also significantly more common in the thick-placenta group than in controls: history of COVID-19 was present in 52/99 cases (52.5%), hypothyroidism in 44/99 (44.4%), prior miscarriage in 37/99 (37.4%), aspirin or anticoagulant use in 32/99 (32.3%), gestational diabetes mellitus in 25/99 (25.3%), and pregnancy-induced hypertension in 7/99 (7.1%). None of these conditions or medication exposures was documented in the control group. Fisher’s exact testing showed statistically significant between-group differences for all evaluated maternal clinical factors, including pregnancy-induced hypertension (*p* = 0.014).

In the thick-placenta group, fetal findings were characterized predominantly by functional abnormalities rather than structural defects (Table 4 and Table 5). Extracardiac dysfunctions were identified in 71/99 cases (71.7%), cardiac dysfunctions in 56/99 cases (56.6%), congenital heart defects in 31/99 cases (31.3%), and extracardiac malformations in 10/99 cases (10.1%). Overall, fetal cardiac or extracardiac dysfunction was present in 68.7% of pregnancies with marked placental thickening. By definition, no fetal structural or functional abnormalities were documented in the control group.

**Figure 3:**
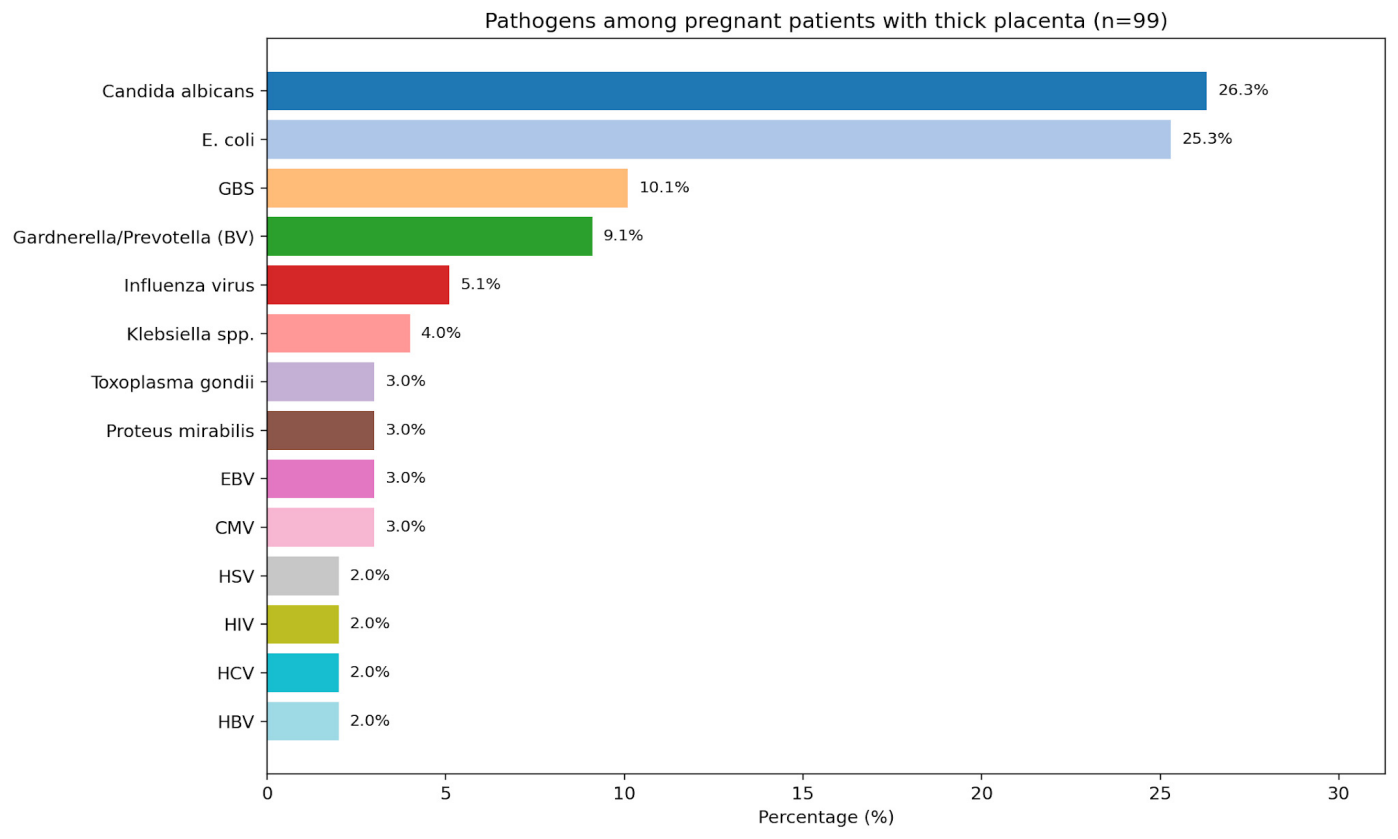
Distribution of pathogens among 99 pregnant patients with a thick placenta. The most commonly identified pathogens were *Candida albicans* (26/99, 26.3%) and *Escherichia coli* (25/99, 25.3%), followed by group B streptococcus (GBS) (10/99, 10.1%) and bacterial vaginosis-associated flora (*Gardnerella*/*Prevotella*) (9/99, 9.1%). Less common pathogens included *Klebsiella* spp., *Proteus mirabilis*, HSV, HBV, HCV, *Toxoplasma gondii*, CMV, EBV, HIV, and influenza virus. Percentages are calculated based on the total study population (n = 99).

The microbiological profile of pregnancies with a thick placenta was dominated by genitourinary pathogens (Figure 3). Candida albicans was identified in 26/99 cases (26.3%) and Escherichia coli in 25/99 cases (25.3%), together accounting for 51.6% of the cohort. They were followed by group B Streptococcus in 10/99 cases (10.1%) and Gardnerella/Prevotella in 9/99 cases (9.1%). Less common pathogens were observed in 5.1% or fewer cases each.

Neonatal outcome data were available for 27 of 99 cases (27.3%) in the thick-placenta group (Table 6) and were not available for the control group; therefore, these data are presented as a descriptive summary of available follow-up information only. No comparative analysis with controls was performed, and no clinical inference regarding neonatal risk should be drawn from these incomplete data.

The mean gestational age at delivery was 38.2 ± 1.9 weeks, with a median of 38 + 5 weeks and a range from 33 + 0 to 40 + 6 weeks. The mean birth weight was 3362.6 ± 800.2 g, with a median of 3810 g and a range from 1480 to 4670 g. Preterm birth before 37 weeks occurred in 5/27 cases (18.5%). Delivery was by cesarean section in 16/27 cases (59.3%), spontaneous vaginal delivery in 9/27 cases (33.3%), and forceps-assisted delivery in 2/27 cases (7.4%). The median Apgar score was 9 at 1 min and 10 at 5 min. Respiratory failure requiring NICU care was documented in 8/27 newborns (29.6%), neonatal jaundice in 11/27 (40.7%), and transient pulmonary hypertension in 4/27 (14.8%). One neonatal death was recorded on day 5 of life. Placental weight was not available in the neonatal dataset.

## 4. Discussion

**Figure 4:**
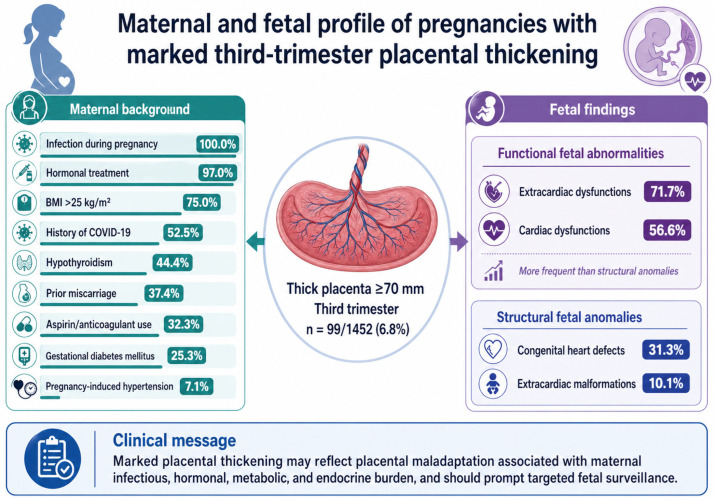
Summary of maternal and fetal findings in pregnancies with marked third-trimester placental thickening. Among 99 singleton pregnancies with a placental thickness ≥70 mm at ≥28 weeks of gestation, the maternal profile was dominated by infection during pregnancy, hormonal treatment, elevated BMI, prior COVID-19 infection, and hypothyroidism. On the fetal side, functional abnormalities were more common than structural anomalies, with extracardiac dysfunctions and cardiac dysfunctions observed more commonly than congenital heart defects or extracardiac malformations.

In this retrospective, gestational-age-matched case–control study, marked third-trimester placental thickening was associated with a distinct maternal and fetal clinical profile (Figure 4). Compared with healthy controls with normal placental thickness, no documented maternal disease, no medication exposure, and no fetal structural or functional abnormalities, pregnancies with a placental thickness ≥70 mm showed a significantly higher burden of maternal infection, hormonal treatment, metabolic disease, endocrine comorbidity, prior miscarriage, and fetal functional abnormalities.

A major strength of the present analysis is the inclusion of a gestational-age-matched control group. Placental thickness may vary with gestational age; therefore, a meaningful comparison requires similar gestational age at examination. In our study, mean and median gestational age did not differ significantly between the control and thick-placenta groups, whereas placental thickness was markedly and significantly higher in the thick-placenta group. This supports the interpretation that the observed placental thickening was not simply a consequence of later gestational age at examination.

The most striking between-group difference was the prevalence of maternal infection. No infections were documented in the clinically healthy control group, whereas infection during pregnancy was recorded in all thick-placenta cases. Although the retrospective design does not establish causality, the magnitude of this difference suggests that infectious or inflammatory processes may contribute to the maternal clinical profile observed in pregnancies with marked placental thickening.

The predominance of Candida albicans, Escherichia coli, group B Streptococcus, and Gardnerella/Prevotella further suggests that genitourinary infection may be a common clinical feature in pregnancies with marked placental thickening. Local or systemic inflammatory signaling may contribute to placental edema, altered vascular permeability, or compensatory villous enlargement, all of which may manifest sonographically as increased placental thickness [2,3].

The higher prevalence of elevated BMI, gestational diabetes mellitus, hypothyroidism, and pregnancy-induced hypertension in the thick-placenta group supports a potential role of maternal metabolic and endocrine dysregulation in abnormal placental growth. Maternal overweight and obesity are associated with chronic low-grade inflammation and altered placental nutrient signaling [3,4], whereas diabetes and thyroid dysfunction may affect placental vascularization, transport function, and trophoblast activity [4,5,6]. In this context, placental thickening may represent an adaptive or maladaptive response to an unfavorable maternal environment rather than an isolated ultrasound finding (Figure 4) [7,8,9,10,11].

Hormonal treatment was reported in nearly all pregnancies with thick placenta and was absent in controls. This finding should not be interpreted as evidence that hormonal treatment directly causes placental thickening. More likely, hormonal treatment acts as a marker of pregnancies already considered high risk because of infertility treatment, threatened miscarriage, prior pregnancy loss, or suspected placental dysfunction. Similarly, aspirin and anticoagulant use likely reflect pre-existing maternal or placental risk rather than an independent causal factor.

The maternal findings should be interpreted together with the fetal profile of the thick-placenta group. Fetal functional abnormalities were more common than structural malformations. Extracardiac and cardiac dysfunctions were common, while congenital heart defects and extracardiac malformations were less common. This pattern suggests that marked placental thickening may identify pregnancies in which maternal disease burden, placental maladaptation, and fetal functional compromise coexist (Figure 4) [12,13,14,15].

The frequency of marked placental thickening in our tertiary referral cohort was 6.8%, which is somewhat higher than the 4.3% incidence reported by Miwa et al. [13] using a gestational-age-adjusted >95th percentile definition. This difference likely reflects our tertiary referral setting, the selected high-risk population, and the use of an absolute threshold of ≥70 mm.

The novelty of the present study lies in the comparison of pregnancies with marked third-trimester placental thickening with a gestational-age-matched healthy control group. Previous studies have mainly examined placental thickness as a predictor of adverse fetal or neonatal outcome, whereas the maternal infectious, hormonal, metabolic, and endocrine profile of such pregnancies has been less clearly characterized [1]. By including matched controls, the present analysis strengthens the evidence that marked placental thickening is associated with a specific maternal risk profile rather than gestational age alone.

This study has several limitations. Its retrospective design and performance in a tertiary referral center introduce potential selection bias. The study population is not representative of the general obstetric population. Patients referred to such centers may have a higher prevalence of maternal comorbidities, fetal abnormalities, and medication exposure. Therefore, the external validity of the present findings is limited, and the results should be interpreted within the context of a selected referral cohort.

Although the inclusion of a gestational-age-matched control group strengthens the analysis, the control group was selected to include clinically healthy pregnancies without documented maternal disease, medication exposure, or fetal abnormalities. As a result, the magnitude of between-group differences in maternal comorbidities, infection history, medication exposure, and fetal findings may have been amplified by study design.

Therefore, the present results should not be interpreted as population-based risk estimates or as evidence that individual maternal factors independently cause placental thickening. The findings are best regarded as descriptive and hypothesis-generating associations observed in a selected tertiary referral cohort. Prospective studies including less selected control groups and appropriate multivariable adjustment are required to confirm these observations.

Another limitation is that gestational-age matching was performed retrospectively by selecting controls with comparable gestational age at examination, rather than by propensity-score matching or another formal matching algorithm. Standardized mean differences were not originally calculated. Therefore, the adequacy of matching should be interpreted cautiously, and the comparison of gestational age between groups should be regarded as descriptive.

In addition, only anterior and fundal placentas with available third-trimester measurements were included, which may reduce generalizability. Placental histopathology was not systematically available, placental weight was not available, for the full cohort. Therefore, the present study cannot determine whether sonographic placental thickening corresponded to villous edema, inflammation, vascular lesions, maternal vascular malperfusion, or other histopathological abnormalities. For this reason, mechanistic interpretations should be considered speculative and hypothesis-generating.

Neonatal outcomes were only available for 27 cases, limiting assessment of the clinical consequences of marked placental thickening.

Another limitation concerns placental thickness measurement. Although measurements were performed using a standardized sonographic approach, they were obtained during routine clinical examinations rather than within a prospective reproducibility protocol. Not all cases were independently measured by both operators, the operators were not formally blinded to all clinical information, and intraobserver and interobserver variability were not assessed. Therefore, measurement variability cannot be excluded.

Although infection-related findings were systematically recorded, they represented a heterogeneous composite variable including active infection, asymptomatic colonization, serological or PCR-based findings, and questionnaire-reported history. Therefore, the present study cannot determine the independent contribution of any specific pathogen, infection site, or infectious mechanism to placental thickening. Future prospective studies should use standardized microbiological protocols and separate active infection from colonization and previous infectious exposure.

The absence of multivariable adjustment is an important limitation of this study. Several maternal factors analyzed in this cohort, including infection-related findings, elevated BMI, hypothyroidism, gestational diabetes mellitus, hormonal treatment, and aspirin or anticoagulant use, may overlap clinically and biologically. Therefore, the present analysis cannot determine which of these factors are independently associated with marked placental thickening. In addition, because the control group was intentionally selected to exclude maternal disease, fetal abnormalities, and analyzed medication exposure, several variables showed complete or near-complete separation between groups. This made stable multivariable modeling inappropriate in the present dataset. The observed differences should therefore be interpreted as unadjusted descriptive associations, not as independent predictors or unbiased risk estimates. Future prospective studies including less selected control groups and sufficient numbers of exposed and unexposed pregnancies will be required to perform appropriate multivariable analyses and to identify independent maternal predictors of marked placental thickening.

Neonatal outcome data were available only for a subset of 27/99 thick-placenta cases and were not available for the control group. Therefore, neonatal findings should be interpreted as descriptive follow-up data only. The incomplete availability of neonatal outcomes and the absence of control neonatal data preclude comparative analysis and prevent any reliable inference regarding neonatal risk associated with marked placental thickening.

## 5. Conclusions

In this gestational-age-matched case–control study, marked third-trimester placental thickening was associated with a distinct maternal and fetal clinical profile compared with healthy controls with normal placental thickness. Despite comparable gestational age, maternal age, and maternal anthropometric characteristics, pregnancies with placental thickness ≥70 mm showed significantly higher rates of maternal infection, hormonal treatment, hypothyroidism, gestational diabetes mellitus, aspirin or anticoagulant use, pregnancy-induced hypertension, prior miscarriage, and fetal cardiac or extracardiac dysfunction.

These findings should be interpreted as unadjusted associations observed in a selected tertiary referral cohort. Marked placental thickening may therefore represent a clinically relevant ultrasound finding associated with maternal infectious, endocrine, metabolic, and obstetric burden, rather than an independent marker of placental maladaptation or maternal-fetal risk. Identification of a placental thickness ≥70 mm in the third trimester may justify careful review of maternal infectious, endocrine, metabolic, and obstetric history, together with targeted fetal assessment.

Prospective multicenter studies incorporating standardized maternal assessment, placental histopathology, placental weight, comprehensive microbiological evaluation, and complete neonatal follow-up are needed to determine which maternal factors are independently associated with placental thickening and to clarify the mechanisms linking maternal health, placental morphology, fetal function, and neonatal outcomes.

These findings suggest that marked placental thickening may represent a clinically relevant sonographic finding associated with maternal infectious, hormonal, metabolic, and endocrine burden and should prompt careful maternal assessment and targeted fetal surveillance.

## Figures and Tables

**Table 1:** Reasons for referral to our tertiary referral center for targeted fetal and echocardiographic examination.

Reason for Referral	Comment
Abnormal screening ultrasound findings	Primary referral pathway
Maternal diseases/comorbidities	Including diabetes, hypothyroidism, and infections
Family history of congenital heart disease	Primary referral pathway
Thick placenta as an isolated reason for referral	None

**Table 2:** Standardized Maternal Questionnaire Used in Study Enrollment. This standardized form was completed for each patient referred to fetal echocardiography. It was used to collect structured data on maternal background, comorbidities, medications, and prenatal history.

Patient Initials/ID number	
Last Menstrual Period	DD.MM.YYYY
Estimated Due Date	DD.MM.YYYY
Gestational Age at Exam	[Weeks + Days]
Weight (kg)	[e.g., 66.8]
Height (cm)	[e.g., 153]
Chronic Conditions	Check for: Diabetes/Hypertension/Hypothyroidism/Epilepsy, etc.
Infectious History/infection-related findings during pregnancy	Check for: HSV, HBV, HCV, CMV, EBV, Toxoplasmosis, HIV, influenza, urinary tract infection, vaginal/cervical infection, bacterial vaginosis-associated flora, Candida spp., GBS, Escherichia coli, Klebsiella spp., Proteus mirabilis, etc. Site of infection: genitourinary/respiratory/gastrointestinal/systemic/other Symptomatic: yes/no Clinical symptoms: fever/dysuria/vaginal discharge/cough/other Basis of diagnosis: urine culture/vaginal or cervical swab/sputum culture/serology/PCR/other Pathogen identified: yes/no Pathogen group: bacterial/fungal/viral/parasitic/mixed/unknown Timing: first trimester/second trimester/third trimester/date of diagnosis Diagnostic basis: urine culture/vaginal or cervical swab/other microbiological culture/serology/PCR/medical record/questionnaire report/other Active infection during pregnancy: yes/no/unclear Colonization or positive screening only: yes/no/unclear Questionnaire-reported history only: yes/no
Clinical category	Symptomatic infection/asymptomatic colonization/positive screening swab or culture/serological evidence/PCR-confirmed infection/questionnaire-reported history Symptoms: yes/no Clinical symptoms: fever/dysuria/vaginal discharge/cough/gastrointestinal symptoms/other
Prior Pregnancy Loss	[Yes/No]
Medications in Pregnancy	Treatment administered; type of treatment if available Clexane, Aspirin, Duphaston, Luteina, Progesterone Besins, Utrogestan, etc.

**Table 3:** Study material and clinical characteristics of pregnancies with marked placental thickening (PT ≥ 70 mm) compared to controls.

Parameter	Controls (PT < 70) (n = 99)	Thick Placenta Group (PT ≥ 70) (n = 99)	*p*-Value
Mean GA (weeks)	35.5	35.0	0.662 (Welch *t*-test)
Median GA (weeks)	35.4	36.43	0.340 (Mann–Whitney)
Range GA (weeks)	33.4–38.4	28–40	not analyzed
Mean maternal age (years)	31	31.2	not significant
Mean maternal height (cm)	162	165	not analysed
Mean maternal weight (kg)	73	77.3	not analyzed
Mean maternal BMI (kg/m^2^)	27.6	28.4	not analyzed
BMI > 25 kg/m^2^	60%	75.0%	*p* = 0.05
Mean PT (mm)	46.9 mm	81.4 mm	<0.0001 (Welch *t*-test)
SD PT	11.2	9.7	not analyzed
Median PT	45.7	80.0	not analyzed
Range PT	27.3–68.6	70.0–123.4	not analyzed
Infection during pregnancy	0/99, 0%	99/99; 100.0%	<0.0001, Fisher exact
Hormonal treatment during pregnancy	0/99, 0%	96/99; 97.0%	<0.0001, Fisher exact
History of COVID-19	0/99, 0%	52/99; 52.5%	<0.0001, Fisher exact
Hypothyroidism	0/99, 0%	44/99; 44.4%	<0.0001, Fisher exact
Prior miscarriage	0/99, 0%	37/99; 37.4%	<0.0001, Fisher exact
Aspirin/anticoagulant use	0/99, 0%	32/99; 32.3%	<0.0001, Fisher exact
Gestational diabetes mellitus	0/99, 0%	25/99; 25.3%	<0.0001, Fisher exact
Pregnancy-induced hypertension	0/99, 0%	7/99; 7.1%	0.014, Fisher exact

**Table 4:** Fetal cardiac and extracardiac findings in the thick-placenta group (n = 99) [1].

Finding Category	Brief Definition	n/99	%
Extracardiac dysfunction	Including: amniotic fluid abnormalities, ventriculomegaly, pyelectasis, growth restriction, abnormal Doppler findings.	71	71.7
Cardiac dysfunction	Includes cardiomegaly, myocardial hypertrophy, valve regurgitation, abnormal Doppler flow patterns, arrhythmias, or impaired contractility.	56	56.6
Congenital heart defects (CHDs)	Structural abnormalities of the fetal heart. Detailed diagnoses are listed in Table 5.	31	31.3
Extracardiac malformations (ECMs)	Structural anomalies outside the heart. Detailed diagnoses are listed in Table 5.	10	10.1

**Table 5:** Detailed list of congenital heart defects and extracardiac malformations identified in the thick-placenta group (n = 99). Percentages were calculated using the total thick-placenta group as the denominator (n = 99). CHDs were present in 31/99 fetuses (31.3%); however, 40 CHD diagnostic entries/patterns were recorded because some fetuses had more than one cardiac defect. ECMs were present in 10/99 fetuses (10.1%).

A. Congenital Heart Defects (CHDs):		
Congenital Heart Defect/Diagnostic Pattern	n	% of Thick-Placenta Group
Ventricular septal defect (VSD)	8	8.1
Coarctation of the aorta (CoA)	4	4.0
Hypoplastic left heart syndrome (HLHS)	4	4.0
Transposition of the great arteries (TGA)	4	4.0
Hypoplastic right heart syndrome (HRHS)	2	2.0
Tetralogy of Fallot (TOF)	2	2.0
Tricuspid atresia (TA)	2	2.0
ALCAPA (Anomalous Left Coronary Artery from the Pulmonary Artery)	1	1.0
APVD (Anomalous Pulmonary Venous Drainage)	1	1.0
Aberrant right subclavian artery (ARSA)	1	1.0
Atrioventricular septal defect (AVSD)	1	1.0
CoA + VSD + TA	1	1.0
Double-outlet right ventricle (DORV) + TGA	1	1.0
DORV + VSD	1	1.0
Ebstein’s anomaly	1	1.0
HLHS + left persistent superior vena cava (LSVC) + VSD	1	1.0
HRHS + aortopulmonary window (APW)	1	1.0
Left persistent superior vena cava	1	1.0
Pulmonary atresia (PA) + tricuspid dysplasia (TD)	1	1.0
Pulmonary stenosis (PS)	1	1.0
TD + VSD	1	1.0
**B. Extracardiac Malformations (ECMs):**		
**Extracardiac Malformation**	**n**	**% of Thick-Placenta Group**
Polycystic kidney	2	2.0
Absent ductus venosus	1	1.0
Double kidney	1	1.0
Gastroschisis	1	1.0
Heterotaxy	1	1.0
Pulmonary hypoplasia	1	1.0
Renal hypoplasia	1	1.0
Skeletal-system defect	1	1.0
Umbilical hernia	1	1.0

**Table 6:** Descriptive summary of neonatal outcomes in cases with available postnatal data from the thick-placenta group (n = 27/99).

Neonatal Outcome	Value
Cases with available neonatal outcome data	27/99 (27.3%)
Gestational age at delivery, mean ± SD	38.2 ± 1.9 weeks
Gestational age at delivery, median, range	38 + 5 weeks, 33 + 0 to 40 + 6 weeks
Birth weight, mean ± SD	3362.6 ± 800.2 g
Birth weight, median, range	3810 g, 1480–4670 g
Apgar score at 1 min, median, range	9, 3–10
Apgar score at 5 min, median, range	10, 5–10
Cesarean section	16/27 (59.3%)
Spontaneous vaginal delivery	9/27 (33.3%)
Forceps delivery	2/27 (7.4%)
Preterm birth < 37 weeks	5/27 (18.5%)
Respiratory failure requiring NICU care	8/27 (29.6%)
Neonatal jaundice	11/27 (40.7%)
Transient pulmonary hypertension	4/27 (14.8%)
Neonatal death	1/27 (3.7%); death on day 5 of life
Stillbirth	Not recorded in the available neonatal dataset
Placental weight	Not available
Birth weight ≤ 3000 g/suspected growth restriction or low birth weight category	13/27 (48.1%)
Birth weight ≥ 3800 g/high birth weight category	14/27 (51.9%)
Birth weight ≥ 4000 g	6/27 (22.2%)

## Data Availability

The original contributions presented in this study are included in the article. Further inquiries can be directed to the corresponding author.
